# A multilevel analysis of the determinants of handwashing behavior among households in Eswatini: a secondary analysis of the 2014 multiple indicator cluster survey

**DOI:** 10.4314/ahs.v20i4.58

**Published:** 2020-12

**Authors:** Maswati S Simelane

**Affiliations:** Department of Statistics and Demography. Faculty of Social Sciences. The University of Eswatini

**Keywords:** Handwashing, factors, Eswatini, households, multilevel logistic regression

## Abstract

**Introduction:**

Handwashing with soap has received considerable attention due to its importance in the prevention and interruption of the transmission of diseases. Regardless of the positive effects of handwashing with soap, developing countries still have a low rate of handwashing.

**Objective:**

The study aimed to determine the individual, household and community-level factors associated with handwashing behavior among households in Eswatini

**Methods:**

Using the Eswatini Multiple Indicator Cluster Survey conducted in 2014, a secondary analysis was done of the households surveyed. A total of 1,520 households nested in communities with complete data on handwashing practices were included in the analysis. Univariate, bivariate analysis and multivariate multilevel logistic regression were used to establish the factors that were associated with handwashing behavior.

**Results:**

The prevalence of handwashing among households was 56% in 2014. Households whose heads were aged 35–54 and 55 years and older were more likely to practice handwashing (AOR=1.88, 95% CI:1.39, 2.54); and (AOR=1.77, 95% CI: 1.205, 2.62) compared to those aged 15–34 years. Households with a pit latrine or no toilet facility at all, were less likely to practice handwashing (AOR=0.24, 95% CI: 0.17, 0.35); (AOR=0.28, 95% CI: 0.11, 0.71) respectively compared to those with a flush toilet. Region of residence was a community-level variable associated with lower odds of handwashing, with those from the Hhohho (AOR=0.22, 95% CI: 0.14, 0.35) and Manzini region (AOR=0.42, 95% CI: 0.27, 0.67) compared to Lubombo region. Households from communities where access to mass media was high were more likely to practice handwashing (AOR =1.47, 95% CI: 1.05, 2.03) compared to those from communities where access to mass media was low

**Conclusion:**

Households headed by young adults, with pit latrine or no toilet facility at all and lived in the Hhohho and Manzini regions and with low access to mass media, should be targeted for interventions aimed at improving handwashing practices.

## Introduction

Developing countries are infested by diarrheal morbidity and mortality. Globally, approximately 6.3 million children died, and over 500 thousand deaths were due to diarrhea in 2013^1^. In 2016, globally, diarrhea was the fifth cause of death among under-five children, while in Africa, it was the 3^rd^ major cause of morbidity and mortality^2^, ^3^. The variation in child infections and mortality due to preventable diseases such as diarrhea and acute respiratory infections (ARIs) and novel coronavirus (COVID-19) could be attributed to the difference in the environment, quality of food, poor water, and sanitation^4^, ^5^.

Handwashing with soap (HWWS) has received considerable attention due to its importance in the prevention and interruption of transmission of viruses and bacteria causing diarrhea, common flue, ARIs, pneumonia, and (COVID-19), hence reducing the magnitude of diseases^5^,^6^. Handwashing can reduce diarrheal diseases by up to 42% and ARIs by slightly above a third (34%)^7^. Therefore, programs and interventions that campaign for HWWS are considered cost-effective. However, globally less than 20% practice HWWS after fecal contact^8^.

The practice of HWWS is founded on the global agenda of Sustainable Development Goals (SDGs), especially target 6.2 of goal 6, which aims to achieve access to adequate and equitable sanitation and hygiene for all and end open defecation, paying special attention to the needs of women and girls and those in vulnerable situations by 2030^9^. Research has shown that households with the availability of handwashing material were more likely to have improved HWWS and reduced infections^10^,^11^. A meta-analysis study showed 31% and 21% of a reduction in gastrointestinal and respiratory diseases, respectively, with improvement in handwashing behavior^7^. A cluster randomized control trial conducted in Tanzania, Vietnam, Peru, and Senegal showed 2 to 2.4 odds of health caregivers washing their hands when the place of handwashing, water, and soap was available^12^. In Bangladesh, a cross-sectional study showed that households with a place of handwashing had a significantly lower likelihood of having reported cough or difficulty in breathing^13^.

Even though several studies have been conducted in other countries to investigate the factors associated with handwashing behavior, they applied a single-level analysis^10^, ^11^, ^13^. Single level methods of analysis are limited compared to multilevel analysis that can control for the clustering of handwashing behavior across communities. Research has shown that individual-level factors cannot adequately explain the health of an individual; hence a new approach called eco-epidemiology should be applied to understand the community level causal pathways of public health outcomes^14^, ^15^.

Regardless of the importance of HWWS as a public health intervention, few studies have been conducted in Sub-Saharan Africa (SSA) that investigated the correlates of handwashing behavior let alone the application of multilevel approaches. In Eswatini, only descriptive reports 16, 17, provides baseline information for HWWS, which are limited to provide reliable recommendations for programming for improved HWWS. In 2014, there were 67.5% of households that had soap or other cleansing agents for hand washing in Eswatini^17^. Therefore, the study aimed to identify the fixed effects (measures of association) and ascertain the random effect (measures of variation) of handwashing behavior to inform policy and programming.

## Methods

### Study design and data source

The study adopted a secondary cross-sectional analysis using the 2014 Eswatini Multiple Indicator Cluster Survey (EMICS). The MICS is an international initiative that is nationally representative of the population and is utilized to assist countries in monitoring and tracking indicators on children, women, and men's health and development. MICS uses standardized structured survey questionnaires administered through face to face interviews at the household level^18^.

### Sampling design and study samples

The 2014 EMICS adopted a two-staged sampling strategy that involved systematic sampling of enumeration areas (EAs) and randomly selecting 15 households from each EA^17^. The MICS is hierarchical with households nested in the communities (EAs). Sampling was done to be representative of the four regions in Eswatini, which are Hhohho, Manzini, Shiselweni, and Lubombo. To solicit information on handwashing behavior, household heads were interviewed, and a 95.2% response rate was achieved in the 2014 survey.

In the survey, there were 5,211 households selected; however, 4,865 (93.4%) completed the interview. Out of the households that were interviewed, only 1,809 had a place for handwashing observed during the household interview; 1,533 had water available in the household, and 272 did not have water in the household. The total number of households included in the analysis were 1,520.

### Study Variables

**Outcome variable:** The study outcome variable is handwashing behavior. The household was deemed to be practicing handwashing if it had a place for handwashing, and water was available with soap/ detergent at the specific place coded as 1. In contrast, those that had a place for washing hands but without water and soap or detergent in the particular place were coded 0. These were assessed through rapid observation during the household interview and is recommended to be the most efficient methods in community surveys 19.

**Explanatory variables:** To understand the determinants of handwashing behavior, several individual, households, and community-level factors were utilized^20^–^22^. The individual-level factors included age of the household head (15–34, 35–54, 55 and above), sex of the household head (male, female), and education level of the household head (no education, primary, secondary, high school, tertiary). The household-level factors were household size (1–3, 4–6, 7 and above), listening to the radio (yes, no), watch television (yes, no), source of water supply (improved, unimproved), toilet facility in the household (flush, pit latrine, no facility), and household wealth index (poorest, poor, middle, rich, richest). The household wealth index was developed using the principal component analysis (PCA)^23^. The household wealth index was already calculated in the MICS dataset and was categorized into five quartiles, namely the poorest, poor, middle, rich, and richest^17^. Wealth indices use information about household durable assets, such as housing materials, toilet or latrine access, phone ownership, or agricultural land and livestock, which are regularly collected in most household surveys to create an index of household wealth^23^. The community-level factors were the place of residence (rural, urban), the region of residence (Hhohho, Manzini, Shiselweni, Lubombo), community poverty (low, high), the proportion of households with lower than secondary education level (low, high), the proportion of households with unimproved water sources in the community (low, high), and the proportion of households with access to mass media in the community (low, high). The community variables were derived from the individual and household level variables and aggregated at the EA, categorized as low and high guided by the literature^24^, ^25^.

## Statistical analysis

Stata 15 was used for the analysis. To account for the complex MICS design, weights were applied through the svy command. Frequencies and percentages were used to describe the study sample. Bivariate analysis with chi-square test statistics was performed to test the independence of the distribution between the independent variables and handwashing behavior. The vector inflation factor (VIF) was used to test for strongly correlated explanatory factors, and no factors were strongly correlated to each other (see Table S1). The command melogit was used to fit the multilevel logistic regression to identify the factors that were significantly associated with handwashing behavior. Multilevel analysis was considered appropriate to account for the hierarchical nature of the MICS and to be able to estimate community-level effects on the outcome variable^26^. A two-level multilevel model was used to report the random effects (measures of variation) and fixed effects (measures of association) of the model. This implies that households (level 1) were nested in communities (level 2). The study specified five models: model 1: (empty model) included only the handwashing behavior to assess the variance across communities. Model 2: included only the individual factors to ascertain how much of the variance was explained by the individual-level factors alone. Model 3: included household-level factors to ascertain how much of the variance was explained by the household level factors alone. Model 4: included only the community-level factors to ascertain how much of the variance was explained by the community level factors alone and model 5: included individual, household and community-level factors in one model to ascertain how much of the variance was explained by the individual, household and community-level factors combined. The fixed effects results were reported as adjusted odds ratios (AOR) at a 95% confidence interval (95% CI). To indicate the variation of handwashing behavior at the community level, the intraclass correlation coefficient (ICC), and the proportion change in variance (PCV) were used. To test for model fitness, the Akaike information criterion (AIC) was used. The model assumptions were assessed using level 1 and level 2 residuals produced during the modeling process.

## Results

Table 1 shows the distribution of the sample. Of the total sample (1,520) of households included in the analysis, almost half (47.1%) were headed by persons aged 35–54 years, while about three in ten (30.9) were aged 15–34 years. A majority of the households (61.8%) had males as heads. Just over a quarter (29.1%) of the households had their heads having a tertiary level of education. Over half (57.6%) of the households were classified under the richest wealth quartile. As for household size, about 67% of the households had 1–3 members, while 28.7% had 4–6 members. Only 2.1% of the households had no toilet facility. A majority (61.4%) of the households were located in urban areas. Only 6.1% of the households were from the Shiselweni region while 25.4% from the Lubombo, 34.9% from Manzini, and 33.5% from the Hhohho region. A majority (77.1%) of the households were located in communities with low poverty. The study sample was almost equally distributed by the proportion of households with lower than secondary education in the community and proportion of households with access to mass media in the community, the proportion of households with unimproved water sources in the community, and proportion of households with access to mass media in the community.

**Table 1:** Descriptive statistics of the study sample

Characteristics	Weighted (n=1,520) n (%)
**Age of HH in years**	
15–34	449 (30.9)
35–54	706 (47.1)
55 and above	365 (22.1)
**Sex of HH**	
Male	924 (61.8)
Female	596 (38.2)
**Education level of HH**	
No education	152 (9.3)
Primary	272 (17.1)
Secondary	303 (19.7)
High school	362 (24.7)
Tertiary	431 (29.1)
**Household size**	
1–3	874 (60.7)
4–6	453 (28.5)
7 and above	193 (10.7)
**Household wealth index**	
Poorest	44 (2.2)
Poor	116 (5.9)
Middle	207 (11.2)
Rich	337 (23.1)
Richest	816 (57.6)
**Listening to radio**	
Yes	1117 (73.0)
No	403 (27.0)
**Watch television**	
Yes	1076 (72.0)
No	444 (28.0)
**Source of Water Supply**	
Improved	1330 (92.6)
Unimproved	144 (7.4)
**Toilet facility**	
Flush	826 (58.6)
Pit latrine	653 (39.3)
No facility	41 (2.1)
**Place of residence**	
Rural	724 (38.6)
Urban	796 (61.4)
**Region**	
Hhohho	590 (33.5)
Manzini	418 (34.9)
Shiselweni	177 (6.1)
Lubombo	335 (25.4)
**Community poverty**	
Low	1079 (77.1)
High	441 (22.9)
**Proportion of households with lower than secondary** **education level**	
Low	763 (50.5)
High	757 (49.5)
**Proportion of households with unimproved water sources in** **the community**	
Low	1142 (79.2)
High	378 (20.8)
**Proportion of households with access to mass media in the** **community**	
Low	771 (48.2)
High	749 (51.8)

Notes: *HH: household head*

### Relationship between each explanatory variable and handwashing behavior

Table 2 shows the relationship between each explanatory variable and household handwashing behavior. The overall prevalence of handwashing behavior among households was 56% (95% CI: 52.9, 59.0). The practice of handwashing was higher, 52.3% among households whose heads were aged 35–54 years, with tertiary education (36.8%), p<0.001. The practice was more common (61.6%) among households with 1–3 members, while only 9.1% of households with 7 and more members practiced handwashing, p<0.001. A majority (71.6%) of the households that practiced handwashing were classified under the richest wealth quartile vs. only 1.3% among the poorest, p<0.001. This study further showed that handwashing behavior was significantly higher (69.1%) among households in rural areas, p<0.001. Regionally, the Manzini region had a significantly higher proportion of households (34.0%) that practiced handwashing behavior, p<0.001. There was also a significant difference by households that watch television, type of toilet facility, community poverty, the proportion of households with unimproved water sources in the community, and the proportion of households with lower than secondary education level, all p<0.001.

**Table 2:** The relationship between factors associated with handwashing behavior

	Place for handwashing with soap		
Characteristics	Yes (%) (Weighted)	No (%) (Weighted)	p-value
**Total**	809	701	
**Prevalence**	56 (95%CI: 52.9, 59.0)	44(95%CI: 41.0, 47.1)	
**Household head age**			<0.001
15–34	209 (26.2)	240 (36.7)	
35–54	427 (52.3)	279 (40.5)	
55 and above	183 (21.5)	182 (22.8)	
**Sex of the HH**			0.111
Male	513 (62.6)	411 (60.7)	
Female	306 (37.4)	290 (39.3)	
**Education level of HH**			<0.001
No education	68 (7.9)	84 (11.2)	
Primary	120 (13.6)	152 (21.5)	
Secondary	139 (16.9)	164 (23.4)	
High school	189 (24.9)	173 (24.6)	
Tertiary	303 (36.8)	128 (19.3)	
**Household size**			<0.001
1–3	482 (61.6)	392 (59.6)	
4–6	256 (29.3)	197 (27.5)	
7 and above	81 (9.1)	112 (12.9)	
**HH wealth index**			<0.001
Poorest	15 (1.3)	29 (3.4)	
Poor	42 (3.8)	74 (8.6)	
Middle	70 (7.2)	137 (16.3)	
Rich	131 (16.2)	255 (39.8)	
Richest	561 (71.5)	255 (39.8)	
**Listen to radio**			<0.001
Yes	641 (76.1)	479 (69.1)	
No	180 (23.9)	224 (30.9)	
**Watch television**			<0.001
Yes	642 (78.6)	437 (63.5)	
No	179 (21.4)	266 (36.5)	
**Source of Water Supply**			0.420
Improved	73 (7.0)	71 (7.9)	
Unimproved	746 (93.0)	630 (92.1)	
**Toilet facility**			<0.001
Flush	591 (75.9)	235 (36.6)	
Pit latrine	213 (22.9)	440 (60.1)	
No facility	15 (1.2)	26 (3.2)	
**Place of residence**			<0.001
Rural	490 (69.1)	306 (51.8)	
Urban	329 (30.9)	395 (48.4)	
**Region**			<0.001
Hhohho	248 (26.5)	342 (42.5)	
Manzini	223 (34.0)	195 (36.0)	
Shiselweni	107 (6.1)	70 (6.1)	
Lubombo	241 (33.2)	94 (15.4)	
Community poverty			<0.001
Low	633 (82.2)	446 (70.5)	
High	186 (17.8)	255 (29.5)	
**Proportion of households** **with lower than secondary** **education level**			<0.001
Low	453 (54.4)	310 (45.5)	
High	366 (45.6)	391 (54.5)	
**Proportion of households** **with unimproved water** **sources in the community**			0.131
Low	628 (80.7)	514 (77.3)	
High	191 (19.3)	187 (22.7)	
**Proportion of households** **with access to mass media in** **the community**			<0.001
Low	363 (42.6)	408 (55.3)	
High	456 (57.4)	293 (44.7)	

Notes: p-value <0.05 for a chi-square test; HH: household head

### Multilevel Analysis

In the final model (Table 3), at the individual level, only the age of the household head was significantly associated with handwashing behavior. Even after controlling for household and community level factors, households whose heads were aged, 35–54 and 55 years and older were more likely to practice handwashing, (AOR=1.88, 95% CI:1.39, 2.54) and AOR=1.77, 95% CI:1.205, 2.62) respectively compared to those aged 15–34 years.

**Table 3:** Results of the individual, household and community-level factors associated with handwashing behavior

Variables	Model 1	Model 2	Module 3	Module 4	Module 5
Fixed effects		AOR(95%CI)	AOR(95%CI)	AOR(95%CI)	AOR(95%CI)
**HH level**					
**Age of HH**					
15–34		1			1
35–54		2.08 (1.56, 2.79)*			1.88 (1.39, 2.54)*
55 and above		1.98 (1.36, 2.88)*			1.77 (1.21, 2.62)*
**Sex of HH**					
Male		1			1
Female		0.92 (0.71, 1.19)			0.94 (0.72, 1.21)
**Education level HH**					
No education		1			1
Primary		1.15 (0.72, 1.85)			1.20 (0.74, 1.93)
Secondary		1.32 (0.82, 2.13)			0.91 (0.55, 1.52)
High school		1.91 (1.17, 3.11)*			1.14 (0.67, 1.94)
Tertiary		4.10 (2.49, 6.73)*			1.43 (0.82, 2.52)
**Household-level**					
**Household size**					
1–3			1		1
4–6			1.37 (1.02, 1.84)*		1.24 (0.92, 1.68)
7 and above			1.09 (0.73, 1.65)		0.98 (0.64, 1.49)
**Household wealth index**					
Poorest			1		1
Poor			1.35 (0.53, 3.42)		1.37 (0.55, 3.39)
Middle			1.20 (0.46, 3.12)		1.27 (0.49, 3.31)
Rich			1.25 (0.46, 3.37)		1.40 (0.51, 3.82)
Richest			2.68 (0.93, 7.75)		2.94 (0.98, 8.79)
**Listen to radio**					
Yes			1		1
No			0.74 (0.55, 1.00)		0.76 (0.56, 1.03)
**Watch TV**					
Yes			1		1
No			0.92 (0.63, 1.34)		0.99 (0.67, 1.45)
**Source of Water Supply**					
Improved			1		1
Unimproved			1.94 (1.21, 3.12)*		1.36 (0.81, 2.28)
**Toilet facility**					
Flush			1		1
Pit latrine			0.24 (0.17, 0.34)*		0.24 (0.17, 0.35)*
No facility			0.25 (0.09, 0.64)*		0.28 (0.11, 0.71)*
**Community-level**					
**Place of residence**					
Rural				0.60 (0.40, 0.89)*	1.02 (0.69, 1.52)
Urban				1	1
**Region**					
Hhohho				0.22 (0.14, 0.35)*	0.22 (0.14, 0.35)*
Manzini				0.36 (0.22, 0.58)*	0.42 (0.27, 0.67)*
Shiselweni				0.42 (0.24, 0.75)*	0.59 (0.34, 1.03)
Lubombo				1	1
**Community poverty**					
Low				1	1
High				0.89 (0.58, 1.36)	1.28 (0.82, 1.99)
**Proportion of** **households with lower** **than secondary** **education level**					
Low				1	1
High				0.73 (0.51, 1.03)	0.99 (0.69, 1.41)
**Proportion of** **households with** **unimproved water** **sources in the** **community**					
Low				1	1
High				1.10 (0.71, 1.71)	1.28 (0.80, 2.03)
**Proportion of** **households with access** **to mass media in the** **community**					
Low				1	1
High				1.83 (1.30, 2.57)*	1.47 (1.05, 2.03)*

**Random effects**	Empty	Individual	Household	Community	Final Model

Community Variance (SE)	1.25 (0.24)*	1.18 (0.24)*	0.76 (0.20)*	0.60 (0.16)*	0.35 (0.4)*
VPC=ICC (%)	27.5	26.4	18.7	15.4	9.6
PCV (%)	Reference	5.7	39.3	51.6	72.1
Log likelihood	-978.86	-940.77	-884.03	-946.46	-842.66
AIC	1961.72	1899.54	1794.06	1912.93	1741.33
n	1520	1520	1520	1520	1520

* p<0.05

SE-standard error, ICC-intraclass correlation coefficient, PVC-proportion change in variance, AOR-adjusted odds ratio, AIC-Akaike information criterion, n-sample observations

At the household level, the toilet facility was associated with handwashing behavior. Consistent with model 3, in the final model, households with a pit latrine and no toilet facility at all were less likely to practice handwashing, (AOR=0.24, 95% CI: 0.17, 0.35); (AOR=0.28, 95% CI: 0.11, 0.71) respectively compared to households with a flush toilet, holding other factors constant in the model.

At the community level, even after controlling for the individual and household level factors, households from Hhohho and Manzini regions were less likely to practice handwashing, (AOR=0.22, 95% CI: 0.14, 0.35), and (AOR=0.42, 95% CI: 0.27, 0.67) respectively compared to households located in the Lubombo region. In the final model, households from communities where access to mass media was high were more likely to practice handwashing, (AOR =1.47, 95% CI: 1.05, 2.03) compared to those from communities where access to mass media was low.

The random-effects results were also reported (Table 3). There was a significant random variation in the log odds of handwashing across communities (τ=1.25, p<0.05), as shown in model 1 (Empty model). The intra-community correlation coefficient (ICC) showed that 27.5% of the variance of handwashing could be attributed to community-level characteristics. The variance remained significant even after controlling for individual-level (Model 2), household level (model 3), and community-level factors (model 4). After controlling for individual, household, and community-level characteristics in one model (model 5), it remained significant. The final model (model 5) had an ICC that implied that 9.6% of the variance of handwashing could be attributed to the individual, household, and community-level factors. However, the proportional change in variance in model 5, shows that individual, household, and community-level factors explained about 72.1% of the variance of handwashing behavior across communities. Model 5 had lower AIC revealing that the inclusion of the individual, household, and community-level factors in one model produced a parsimonious model when compared to the other models.

## Discussion

The study found that overall the practice of handwashing was 56% in Eswatini and that several factors were associated with handwashing behavior. The findings showed a 9 points increase in the prevalence of handwashing with soap in 2014 when compared to 47% in 2010 16. The improvement in the handwashing behavior is in line with the SDGs^9^. The Eswatini government and partners have made efforts to initiate programs such as the water, sanitation, and hygiene (WASH) project, which aimed to improve water supply at the household level hence significantly reducing distances traveled and time taken to collect water resulting in improved hygiene such as handwashing^27^. However, the prevalence of handwashing behavior reported in this study is lower than that reported in the 2014 MICS report, which was at 67.5%^17^. This study limited the definition of handwashing behavior if a household had a place for handwashing and water was available with soap/detergent at the specific place while the 2014 MICS report^17^, included all households with soap and other cleansing agents anywhere in the household. This study showed that handwashing behavior is much lower in Eswatini when compared to a study conducted in Indonesia^28^ and Vietnam^29^. The results imply that initiatives on programs being implemented on handwashing need to be continued and sustained to avert the potential transmission of pathogens that results from unhygienic hands.

Evidence from the literature suggests that the older the household head, the more likely the practice of handwashing in the household^30^, ^31^. This study showed that households whose heads were aged 35 years and older were more likely to practice handwashing. A possible justification for this finding could be that, in this study, a majority of older household heads (i.e., 55 years and older) were from the richest households located in urban areas, had improved water supply and soap (see Table 1).

Evidence in the literature reported a positive relationship between education and handwashing behavior^13^, ^30^,^32^. However, this study found no significant association between the education level of the household head and handwashing behavior in the final model. This could be because improved water supply in rural areas is usually a community project, and lack of improved water may be a problem of the entire community rather than individual households 27.

Several studies found a significant association between the type of toilet facility in the household and handwashing behavior^22^, ^33^, for example, in Vietnam, households with improved sanitation were more likely to practice handwashing^22^. This study found that the odds of handwashing were lower for households that had a pit latrine and no facility at all compared to those with a flush toilet. A justification for the findings could be that poor households with no flush toilets are located in rural areas in Eswatini, where there is a poor supply of improved water and soap. A cross-sectional study found that households in rural areas classified under the poorest quartiles were less likely to have improved water sumply^34^.

This study also found that households from the Hhohho and Manzini regions were less likely to practice handwashing when compared to the Lubombo region. The higher odds of handwashing behavior in the Lubombo region are surprising when compared to the other regions, given that it is the least developed, followed by Shiselweni^35^. The possible explanation for this could be due to the effects of the large scale-up of water, sanitation, and hygiene projects. Other studies done elsewhere showed that there was a regional variation in handwashing behavior^29^, ^36^.

Exposure to mass media has been found to play a significant role in the adoption of a positive attitude towards handwashing behavior. In a study done in Kenya, media exposure was associated with the formation of hygiene behaviors, including handwashing^33^. This study found that where the proportion of households with access to mass media was high, the odds of handwashing was high. This could be because mass media is regarded as an effective hygiene promotion strategy that could be a worthwhile addition to other programs on the ground aimed at improving water and sanitation in the communities.

There are some limitations associated with the study. First: social desirability bias could affect the findings since the data was self-reported 37. Second: the temporality of independent factors and handwashing behavior cannot be ascertained due to the cross-sectional nature of the MICS. Third, self-reported handwashing behaviors tend to be overestimated and are not reliable. Indirect observation of the place of handwashing with water in the household may present as a limitation^38^; however, it is one method of measurement that has been considered reliable in the measurement of handwashing behavior 17, 39. The study aggregated the community-level factors at the EA; this may have resulted in some of the households being misclassified.

## Conclusion

This study confirms the relationship between age of the household head, type of toilet facility, region of residence, the proportion of households with access to mass media in the community, and handwashing practices. To achieve remarkable progress on handwashing practices by 2030, intervention should target households headed by young adults, with pit latrines or no toilet facility at all, and live in the Hhohho and Manzini region. This study highlights that individual, household, and community-level factors have a significant role in determining handwashing behavior among households. The study results suggest that handwashing behavior could be improved if interventions consider the individual, household, and community factors that influence handwashing. Therefore, interventions should be integrative of the individual, household and community-based approaches especially in disadvantaged communities

## Data Availability

The Data is available from https://mics.unihttps://mics.unicef.org/surveyscef.org/surveys upon request.
